# QoS Analysis for Cloud-Based IoT Data Using Multicriteria-Based Optimization Approach

**DOI:** 10.1155/2022/7255913

**Published:** 2022-09-07

**Authors:** L. Jayakumar, R. Jothi Chitra, J. Sivasankari, S. Vidhya, Laura Alimzhanova, Gulnur Kazbekova, Bakhytzhan Kulambayev, Alma Kostangeldinova, S. Devi, Dawit Mamiru Teressa

**Affiliations:** ^1^Department of Computer Science and Engineering, National Institute of Technology, Agartala, Tripura, India; ^2^Department of Electronics and Communication Engineering, Velammal Institute of Technology, Chennai, Tamilnadu, India; ^3^Department of Electronics and Communication Engineering, Ultra College of Engineering and Technology, Madurai, Tamilnadu, India; ^4^Department of Information Technology, Saveetha Engineering College Thandalam, Chennai, Tamilnadu, India; ^5^Al-Farabi Kazakh National University, Almaty, Kazakhstan; ^6^Head of the Department of Computer Sciences, C. T. S Khoja Akhmet Yassawi International Kazakh-Turkish University, Turkistan, Kazakhstan; ^7^International Information Technology University, Almaty, Kazakhstan; ^8^Kokshetau University Named Af Sh Ualijhanov, Kokshetau, Kazakhstan; ^9^Department of Computer Science Engineering, Mother Terasa College of Engineering and Technology, Pudukkottai, Tamil Nadu, India; ^10^Department of Chemical Engineering, College of Biological and Chemical Engineering, Addis Ababa Science and Technology University, Addis Ababa, Ethiopia

## Abstract

This work explains why and how QoS modeling has been used within a multicriteria optimization approach. The parameters and metrics defined are intended to provide a broader and, at the same time, more precise analysis of the issues highlighted in the work dedicated to placement algorithms in the cloud. In order to find the optimal solution to a placement problem which is impractical in polynomial time, as in more particular cases, meta-heuristics more or less approaching the optimal solution are used in order to obtain a satisfactory solution. First, a model by a genetic algorithm is proposed. This genetic algorithm dedicated to the problem of placing virtual machines in the cloud has been implemented in two different versions. The former only considers elementary services, while the latter uses compound services. These two versions of the genetic algorithm are presented, and also, two greedy algorithms, round-robin and best-fit sorted, were used in order to allow a comparison with the genetic algorithm. The characteristics of these two algorithms are presented.

## 1. Introduction

The Internet of things (IoT) will open up new possibilities for developing apps that more effectively incorporate the current status of the sector. Due to the proliferation of web services that perform identical tasks, industrial organizations must select the best web services based on their quality of service (QoS) characteristics. In this article, the QoS problem is formulated as a multicriteria goal programming (MCGP) model, and the model is solved using a multipopulation genetic algorithm (MGA). In addition to automatically selecting high-quality web services to combine into composite services, MCGP also searches for composite services that do not fall short of users' QoS expectations by loosening QoS restrictions. A study of empirical data shows that the genetic algorithm outperforms both round-robin and best-fit sorting. Additionally, the studies show that the genetic algorithm can efficiently and scalably address the large-scale QSC problem.

We define three QoS characteristics as the quality evaluation criteria of web services according to the domain application of IoT, which are defined in relation to various QoS attributes for web services published by the W3C working group:

- Execution time (*t*): the average amount of time that passes between when a user sends a request and when the server responds is the service's execution time (*t*).
- Reliability (*r*): the percentage of service requests that are successfully fulfilled determines how reliable a service is. The ratio of successful executions to total service calls is used to calculate it.
- Execution cost (*c*): the fee for using a web service is known as the execution cost.

A collection of component services with logical connections makes up a composite service (or tasks). Various candidate service instances with the same functionality but distinct QoS values might be bound to different tasks. Sequential, loop, parallel, and switch are the most popular service composition workflows.

**Figure 1:**
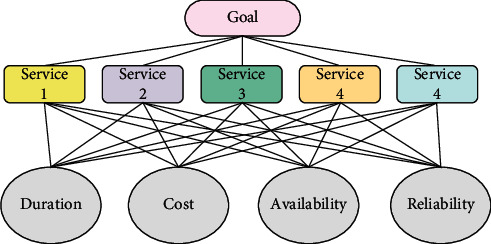
QoS criteria.

In conclusion, researchers try to identify the greatest services to combine into the best composite service for industrial applications. Developing effective strategies to deal with circumstances where no practicable solution can satisfy the overall QoS restrictions is still a crucial task. In order to address this challenge, this work formulates the QoS problem into an MCGP model in order to discover a solution with a lower level of constraint violation. The MCGP model is then solved using a multipopulation genetic method.  Figure 1 shows the QoS criteria.

## 2. Literature Review

The analysis of the SLA proposals of the main current SaaS service providers makes it possible to realize that the inclusion of even one of these parameters directly serves the interests of the service provider [1]. Services, in the research on cloud computing placement algorithms, could allow for further study [2, 3]. For example, if the latent capacity parameter, cited in Oracle's SLA proposal, was used as a metric to be optimized in the placement algorithms, in addition to the energy consumption and response time, this would lead to analysis of an interesting compromise between the reduction in energy consumption, the desired performance at a given moment, and the capacity of the services at the moment, if a peak of use (a sudden increase in the number of requests) occurs. Indeed, taking into account more QoS parameters, assimilated to optimization metrics in placement algorithms aimed at reducing the energy consumed, can lead to choosing a temporary consolidation configuration of virtual machines unfavorable for some metrics, but guaranteeing a high level of performance for other metrics, which are mostly ignored in current studies [4]. The use of several metrics thus makes it possible to have a more complete view of the state of all physical resources, and provides service providers with a more sophisticated analysis possibility as well as a greater number of configuration solutions. In addition, multicriteria optimization also makes it possible to analyze the influence of the parameters with respect to each other. This is performed, in order to estimate the way in which each parameter influences the optimization, to analyze the antagonism caused by the joint use of these metrics, but also to verify their relevance. These different points are not without importance because it is not necessarily easy to select a set of QoS parameters having both real relevance for the analysis of the operation of the system and the joint optimization which, with other parameters, results in a satisfactory compromise. Another interesting advantage of a simultaneous analysis of various cloud QoS settings can be emergence from the a priori undemonstrated properties of greedy algorithms, whose intrinsic behavior does not allow a direct optimization of the analyzed metrics [5, 6].

This work explains why and how QoS modeling has been used within a multicriteria optimization approach. It is also important to remember that in addition to the selected quality of service parameters, reconfigurations of virtual machines as well as DVFS are integral parts of the scheduling problem presented. First, modeling by a genetic algorithm is proposed. This genetic algorithm dedicated to the problem of placing virtual machines in the cloud has been implemented in two different versions [7, 8]. The former only considers elementary services, while the latter uses compound services. This version clearly complicates the resolution of the placement, in particular, because a temporal aspect must be integrated into the resolution. Indeed, the topology of a complex service, however simplistic it may be, induces dependence between the services that the genetic algorithm must take into account to obtain a valid investment solution. These two versions of the genetic algorithm are presented, and also, two greedy algorithms, round robin and best-fit sorted, were used in order to allow a comparison with the genetic algorithm. The characteristics of these two algorithms are presented. It is important to specify here that the version of the algorithm using compound services is presented as preliminary work requiring future work. This version of the genetic algorithm is not used in the other works exposed in the rest of this article but presents a certain interest thanks to the complexities that it raises. The first section of this work presents how the use of several quality of service metrics within scheduling algorithms can both enrich current research studies and improve multiobjectives dedicated to cloud computing, but also highlights the sometimes ignored insight of certain algorithms with more basic behaviors [9–12].

As the importance of quality of service in cloud computing becomes more and more important, this article implements the various types of classifications in cloud computing and includes three major concepts to explain cloud computing. The energy consumption, the response time, and the two other parameters are used to explain the QoS methodology of the entire network services [13–27].

To reduce the power consumption of IoT devices and speed up task execution, Internet of things (IoT) tasks are offloaded to servers at the edge of the network. However, in dangerous terrain or in emergency situations where the network is down, establishing edge servers could be challenging or perhaps impossible. Cloud services are replaced as close to the end user as possible by mobile-edge computing (MEC). This lowers the energy usage and turnaround time delay by allowing the edge servers to carry out the offloaded operations that the users have requested. However, it could be challenging to deploy such edge servers in hostile environments or in disaster zones without a network. Performance improvements are now possible in many domains thanks to recent developments in high-performance computing systems. Surface approximation from a group of points is one of the most significant uses of this improvement. In this study, we suggest a method for creating a surface that approximates an oriented set of samples and is entirely compatible with graphics processing units (GPUs) [28–30].

## 3. Selection of Quality of Service Metrics

Four QoS parameters have been chosen: energy consumption, which makes it possible to take into account environmental problems; the response time, which makes it possible to have a pure performance measure; the robustness, which reassures service providers and users on the probability of being affected by a failure of the system; and the dynamism, which ensures a certain reserve of performance in the event of a traffic peak. In order to be able to measure and evaluate these metrics in the two approaches proposed in this chapter, some modifications had to be made to the definitions of the metrics used compared to those presented.

## 4. Greedy Algorithms

This section presents the greedy algorithms used, described below, in addition to the genetic algorithm presented. These two greedy algorithms are round-robin and best-fit sorted. Their operation is more basic compared to a generic algorithm in that they do not integrate a direct optimization of QoS metrics. However, the multicriteria analysis of their results allows them to interpret their insight in another way.

### 4.1. Round-Robin

The round-robin (RR) algorithm was empirically used for scheduling in networks, with no priority over the chosen destination, when the concept of a virtualized environment did not yet exist. Its use in the context of this chapter may seem rather strange given that the principle of this algorithm does not present any particular intelligence, which would make it possible to take advantage of virtualization and therefore of the notion of virtual machine consolidation, currently commonly used in cloud data centers to minimize energy consumption. Thus, its use in this chapter is analyzed by taking into account several parameters, and not only the energy consumption, which allows a more elaborate analysis to be made to demonstrate its advantages [31–34]. The implemented round-robin sorts the physical machines in ascending order according to their type. That is to say, the algorithm will go through the physical machines of type 0 first, then those of type 1, etc. Due to the intrinsic behavior of the algorithm, this sorting has little importance when a large number of virtual machines must be allocated because virtual machines will still be placed on poorly performing physical machines. But the more the number of virtual machines to allocate decreases, the more interesting this sorting becomes. However, this sort of sorting is not very efficient with round-robin as long as the number of virtual machines to be allocated is greater than the number of physical machines. Indeed, these will be used anyway, whatever their type.

### 4.2. Best-Fit Sorted

The best-fit sorted (BFS) algorithm is better known for its ability to efficiently take advantage of a virtualized environment. Used with sorting performed on the characteristics of physical machines and virtual machines, the BFS obtains good results in terms of energy consumption. The implemented BFS performs two different sorts on physical machines. First, the physical machines are sorted according to their type. Once this first sort is complete, a second sort is performed according to the (decreasing) quantity of free MIPS on each of the physical machines. This not only allows virtual machines to be allocated to the most energy-efficient physical machines, but also attempts to consolidate virtual machines as best as possible.

## 5. Genetic Algorithm

In English, a genetic algorithm (GA) is an optimization meta-heuristic that mimics natural evolution. A genetic algorithm uses a set of individuals, called a population, in which each individual (also called a chromosome) represents a solution to the problem to be solved. The basic principle of a genetic algorithm is to make this initial population evolve, over a certain number of generations, to end up with a population that will contain better chromosomes than the initial ones. From one generation to another, specific operators (genetic operators) are applied to each individual in order to explore new possible solutions. At each generation, following the application of these genetic operators that generate new individuals, all the chromosomes (initial and new) are sorted according to their fitness value. Each individual is assigned a score (fitness value) following the calculation of an objective function. Each individual is therefore evaluated according to this value, and only those chromosomes having obtained a fitness value estimated to be good enough to be part of the generation are selected to constitute the working population of the next generation. Thus, with each generation, the set of operators creating new chromosomes tends to ensure that the population contains individuals representing better solutions. A genetic algorithm is therefore a meta-heuristic, being able to adapt to several types of problems and make a starting population evolve by randomly applying operators, making possible the journey of the solution space [34–37].

Equation (1) is frequently used to assess the fitness of an individual (equivalent to a candidate service composition formed by choosing a specific service instance for each task).

(1) $$\begin{array}{c} F=W_{1}t+W_{2}r+W_{3}c. \end{array}$$

Here, the values of reaction time, dependability, and cost have been combined using aggregation procedures.

The fitness function stated in equation (2) specifically tries to minimize breaches of the QoS performance and the provided QoS constraints for people in the case of no feasible solutions. Algorithm 1.

(2) $$\begin{array}{c} F=W_{1}t+W_{2}r+W_{3}c+W_{4}d_{1}^{*}+W_{5}d_{2}^{-}+W_{6}d_{3}^{+}, \end{array}$$

where 0 < *w*_*i*_ < 1,

(3) $$\begin{array}{c} \sum_{i=0}^{6}w_{i}=1,\\ d_{1}^{*}=t-T_{0},\\ d_{2}^{-}=R_{0}-r,\\ d_{3}^{+}=c-C_{0}. \end{array}$$

### 5.1. Modeling

**Figure 2:**
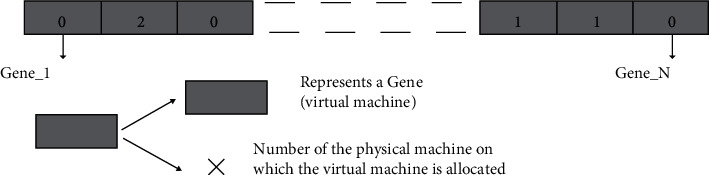
A chromosome is made up of *N* genes.

A genetic algorithm is, by definition, a meta-heuristic that can be adapted to many types of problems. A clear description of the chosen model in the context of this thesis is therefore necessary:

- A chromosome represents a solution for placing virtual machines, as shown in Figure 2
- A gene represents a virtual machine
- The value assigned to a gene represents the number of the physical machine on which the virtual machine has been allocated
- At the same time, the characteristics of virtual machines and physical machines are saved in order to be able to calculate the values of each of the metrics as well as the fitness value of the chromosomes

### 5.2. Operators

**Figure 3:**
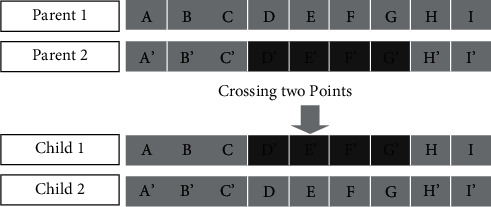
Two-point crossing operator applied to chromosomes.

As mentioned in the introduction, one of the basic principles of an algorithm is to randomly apply operators to the chromosomes of a population in order to form new ones. The best individuals are kept to be part of the next generation, and then the process begins again. The operators used and described are the three typical operators of genetic algorithms:

- The mutation operator, applied to a fixed number of chromosomes, randomly chooses a gene and changes its value. This change in the value of the concerned gene means that the virtual machine represented by this gene has been allocated to another physical machine. Thus, the new chromosome obtained represents a new placement solution and is integrated into the current population.
- The crossover operator, also applied to a fixed number of chromosomes, inverts parts of two chromosomes and thus generates two new solutions. This is performed using two crossing points. An illustrated example of this operator is given in Figure 3.
- The role of the selection operator is to reduce the number of individuals present in the population, temporarily enlarged by the execution of the two operators above, in order to keep the best of them, and thus restore the population to its original size.

### 5.3. Chromosome Validity

An important process of a genetic algorithm is to ensure that the solutions found, following the application of operators, are valid solutions. In other words, the solutions found must respect the constraints of the model of the problem to be solved. For the genetic algorithm used here, this consists in verifying that each solution respects the maximum utilization rate of each resource (CPU and memory). If the check infers that a chromosome does not meet these constraints and is therefore not valid, then it is simply deleted from the current population and a new individual is generated.

### 5.4. Stop Criterion

The termination of a genetic algorithm can be decided in two different ways:

- By defining an improvement threshold that makes it possible to compare the best chromosome of the current generation with the best chromosome of the previous generation. If the difference between these two chromosomes is less than the defined threshold, then the GA is stopped, considering that the improvement between two consecutive generations is not important enough that it is worth continuing the process.
- After a fixed number of generations in the algorithm studied here, the first solution could not be used. Indeed, as explained in the next section, chromosomes are evaluated according to a normalized value. This depends, among other things, on the mean values of each of the metrics (calculated for all individuals in the population). These averages are necessarily different between each generation. Thus, the fitness values of the chromosomes are not comparable between successive generations. Therefore, the second solution was adopted.

### 5.5. Fitness Metrics and Values

Each metric calculation gives a value in an interval intrinsically linked to the relevant metric. In order to be able to calculate correctly the value of the objective function, involving a set of metrics whose values are not included in the same intervals, a normalization of each of these metric values must be applied. This consists in calculating, for each metric, a value called normalize. These values are therefore comparable, and it is then possible to add or subtract them from each other in order to obtain a normalized fitness value. The normalization method used here is the “Center-Reduce” method, whose formula gives a set of values whose mean is 0 and a variance of 1.

(4) $$\begin{array}{c} \vartheta_{cr}=\frac{v-\mu }{\sigma }, \end{array}$$

with *v* the value of the metric to be normalized, *μ* the mean of the metric over the entire population and the standard deviation. Thus, the normalized value of fitness is equal to a linear formula integrating all these normalized values. This therefore makes it possible to compare accurately and precisely each chromosome belonging to a generation according to their fitness value.

The objective function used in this genetic algorithm is as follows:

(5) $$\begin{array}{c} F_{\text{obj}=a_{1}E+a_{2}T_{\text{Resp}}+a_{3}R-a_{4}D}, \end{array}$$

where *a*_1_, *a*_2_, *a*_3_, and *a*_4_ are the respective coefficients of energy (*E*), response time (*T*_Resp_), robustness (*R*), and dynamism (*D*), respectively. These coefficients can be modified (increased or decreased) to benefit the optimization of one or more metrics. In this equation (5) of the objective function, the energy, the response time, and the robustness are metrics to be minimized, unlike the dynamism metric, which must be maximized.

### 5.6. GA Version: Allocation of Elementary Services

**Figure 4:**
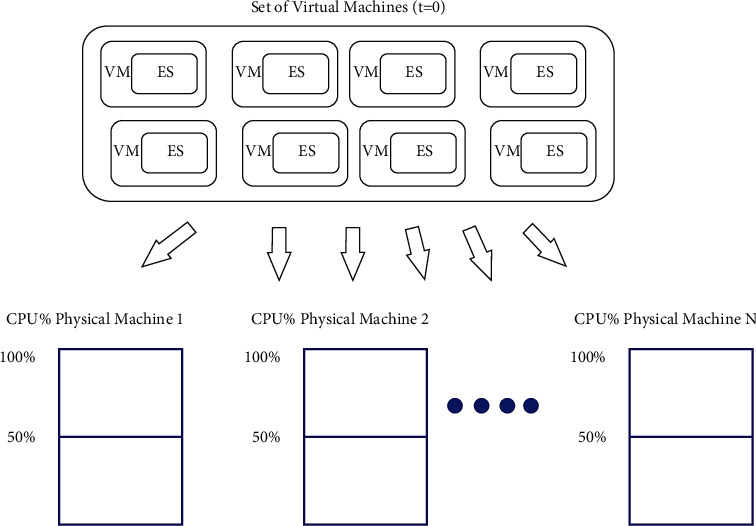
Illustration of the elementary service allocation problem.

This first version of the genetic algorithm only involves the allocation of independent elementary services, each executed in a dedicated virtual machine. Each virtual machine therefore represents an elementary service whose start date is *t* = 0. As illustrated in Figure 4, the solution to the problem consists of finding a placement of the set of virtual machines on the available physical machines while optimizing the QoS metrics.

It is obvious that this version of the genetic algorithm solves the problem of the allocation of very simplified services compared to real cloud services. However, this version of simplified service allocation allows analysis to be focused on optimizing the various selected QoS metrics [38–42]. Indeed, using this genetic algorithm as a placement meta-heuristic makes it possible to evaluate both the quality of its optimization but also to demonstrate the impact of metrics on top of each other, highlighting the advantage of using a multicriteria approach for service allocation purposes. It is this version of the genetic algorithm that is used in the rest of the work presented. The analysis of the impact of multicriteria optimization is discussed in detail in the section below.

### 5.7. GA Version: Allocation of Compound Services

A second version of the genetic algorithm using compound services (*Sc*) has also been explored. A composite service is a set of elementary services (an elementary service is executed by one and only one virtual machine). In a general case, the topology of a compound service is a DAG. This makes it possible to define the dependency relationships between the elementary services and thus, represent the execution order of each of them. One of the main differences from the version presented in the previous section is that the use of compound services brings a temporal aspect to the execution of virtual machines.

### 5.8. Topology

**Figure 5:**
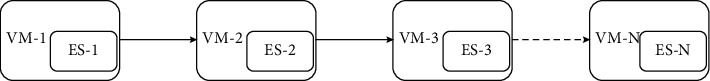
Illustration of the topology of a compound service.

For this first version of the genetic algorithm using compound services, a very simple topology has been chosen (illustrated in Figure 5).

However, this topology makes it possible to introduce a dependency between the elementary services of a composite service, resulting in different departure dates between each elementary service as well as a communication time between two consecutive elementary services. In addition, a virtual machine can only belong to a single compound service.

### 5.9. Generation of Compound Services

All virtual machines are used to create a number of compound services. The size of a composite service has been set between 2 and 10 (chosen randomly when it is created), and the virtual machines that compose it are also chosen randomly. Using the configuration of 400 virtual machines/110 physical machines, this leads to having a number of services between 40 and 200 different sizes. The communication time between two elementary services is fixed at 1 second.

## 6. Calculation of Metrics

Despite these numerous simplifications brought to the configuration of this GA, taking into account the dependencies between the execution dates of virtual machines very clearly complicates the calculation of QoS metrics. Indeed, in order to be able to calculate the total energy consumed for the execution of all these composite services, each start and end date must be known. These are variable depending on the reconfigurations and the composite services to which they belong. The optimization of the energy consumption metric is therefore much more complex to implement. Regarding the response time metric, this always represents the total execution time, here, determined by the termination date of the last elementary service of the last service compound. Its computation is a little finer because the slowing down of one of the virtual machines in a compound service must have an impact on the execution of the virtual machines that run afterward.

In order to clarify the progress of this version of the GA, everything that has been mentioned above is operational; the new issues mentioned below remain to be clarified.

The optimization of the other two metrics still raises new questions. Indeed, since the dynamism and the robustness are being in themselves and metrics are a property of the system not dependent on time, their evaluations in this version of the GA must be submitted. Several solutions are therefore possible:

- Optimize their average value over the total execution time
- Optimize their maximum value reached
- Optimize their value so that it never drops below a certain threshold

Whatever the solution adopted, this implies an evaluation of the values of these metrics at each start-up and termination of virtual machines in order to take into account the evolution of the system load during this time.

This version of the genetic algorithm involves an insignificant number of new constraints and very interesting difficulties, which raise numerous allocation problems. In addition, the use of composite services allows one to come a little closer to the allocation constraints of real cloud services. However, many questions still remain open, and the study of this version of the genetic algorithm is still part of the research work currently underway [36, 37].

### 6.1. Multiobjective Optimization by the GA

The genetic algorithm solves the problem of placing virtual machines with the aim of optimizing the metrics integrated into the computation of its objective function. Each individual of the working population is evaluated according to its value (fitness value). The chosen solution is therefore the individual who will have received the best overall result. The advantage of the genetic algorithm is that it quite easily allows adding or removing parameters to be taken into account when evaluating a population. Moreover, the solution of the problem is carried out in a reasonable time due to the property of the genetic algorithms to quickly converge towards a solution which they consider to be the best. The disadvantage of the genetic algorithm is that it gives a placement result for a given starting situation. That is, it calculates each of the metrics for this situation at the instant *t* = 0 and ignores the time aspect. Of course, the termination dates of each virtual machine are taken into account in the metric calculations, but the optimization is carried out from the starting placement (different according to the chromosomes) and is not questioned as the number of virtual machines still running decreases. This section highlights the advantages of a multiobjective optimization, taking into account four QoS metrics simultaneously compared to a placement focused on a single parameter. For this, five versions of the genetic algorithm have been generated; the first four correspond to the optimization of a single metric, and the last one optimizes equitably the four metrics taken into account. This therefore leads to having the following five GAs:

### 6.2. Configuration

Given the complexity of the allocation problem handled by the GA, it is not uncommon for allocation solutions that it generates to be invalid. That is to say that the proposed placement does not respect the constraints of memory and /or CPU. In this case, the chromosome is rejected and the genetic algorithm generates a new one. If this is repeated too often, the creation time of the starting population, as well as the working population of each generation, can very quickly become extremely long or even never finish (in the case where the placement constraints are too heavy). With 110 physical and 400 virtual machines, the allocation constraints are reasonable but already have a rather heavy impact on the creation of the initial population. Indeed, to find 1500 valid individuals, the GA generates on average around 240,000 invalid chromosomes. With such a ratio between the number of chromosomes desired for the initial population and the number of invalid individuals, the creation time of the initial population is about 4 seconds. This equates to approximately 10% of the total GA execution time. The number of 1500 was therefore considered as a good compromise between a number of starting individuals not too low in order to have all the same a chance that the starting population is composed of interesting individuals and a reasonable generation time. Although there is no universal setting for the use of an algorithm, starting values giving good results are shown in Table 1.

Except for the value of the number of crossings, the values adopted for the genetic algorithm are higher than these theoretical values: a working population of 120 individuals, 75% crossing, 83% mutation, and a much higher number of 600 generations. These values were chosen to both have a reasonable total resolution time of the GA (about 40 seconds) and an efficient solution search given the complexity of the problem allocation to resolve. In the performance evaluation phases of this GA, it was found to be more efficient to use a reasonable working population size and a large number of generations. This is why the choice of a number of 120 individuals, allowing a reasonable processing time for each generation, and a number of 600 generations favouring optimization between generations, has been made. Regarding the crossings, a percentage corresponding to the theoretical value was adopted because the crossing process showed that it would generate a large number of invalid solutions. There was therefore no point in increasing its number. Conversely, the mutation operator has proven to be very efficient in generating good solutions. Applied to the problem studied here, this operator corresponds to migrating a virtual machine out of 100 different individuals at each generation.  Table 2 shows the universal setting for the use of an algorithm.

### 6.3. Optimization Configurations

The genetic algorithm has been broken down into 5 different versions. Each of them applies a different optimization of the metrics:

- GA *E* optimizes energy
- GA *T*_Resp_ optimizes the response time
- GA *R* optimizes the robustness
- GA *D* optimizes the dynamism
- GA All optimizes all these metrics simultaneously and decently

The values of the coefficients corresponding to each version of the GA are summarized in Table 3.

The coefficient values shown in Table 3 for each version of the GA correspond to the weights assigned to the coefficients (*a*_1_, *a*_2_, *a*_3_, and *a*_4_) used in the calculation of the objective function. When a value equal to 0 is applied to the coefficient of a metric, then that metric is totally ignored. Thus, for the four mono-optimization versions (GA *E*, GA *T*_Resp_, GA R, and GA *D*), the value of 1 allows only the desired metric to be optimized. Any other value greater than 0 and different from 1 would have exactly the same effect on optimization. For the multiobjective version (GA All), the value of 1 (when it is not 0) applied to the different coefficients has no meaning in itself. Indeed, although we are dealing here with a multicriteria optimization concerning metrics with different units, a standardization method was applied to each of them. Once the calculation of the objective function uses standardized values of metrics, then the absolute value of each of the coefficients is irrelevant. Indeed, it is only the difference in the values applied to them that can favour one metric over others. In other words, the same positive value different from 1 could have been applied to all the coefficients of the GA All, giving equal weight to each metric considered in the calculation of the objective function.

### 6.4. Comparison of Optimization Results

This section compares and critiques the different optimization results obtained with the different versions of the genetic algorithm.

**Figure 6:**
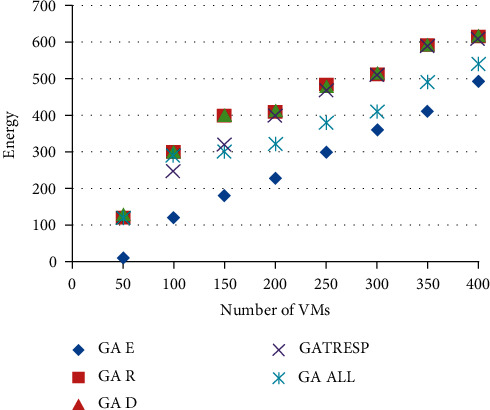
Comparison of results on the energy metric.

**Figure 7:**
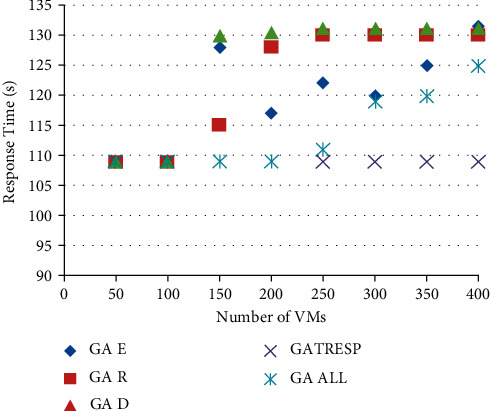
Comparison of the results on the response time metric.

**Figure 8:**
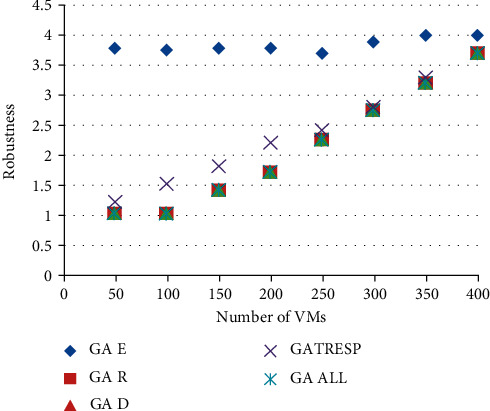
Comparison of results on the robustness metric.

**Figure 9:**
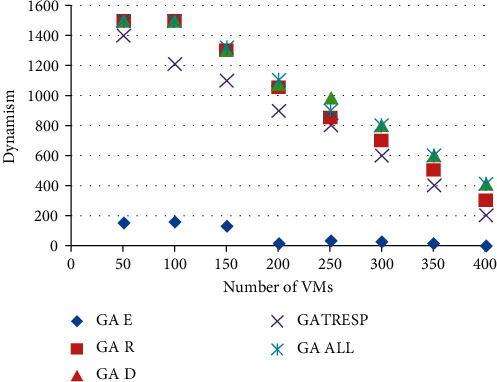
Comparison of results on the dynamism metric.

**Figure 10:**
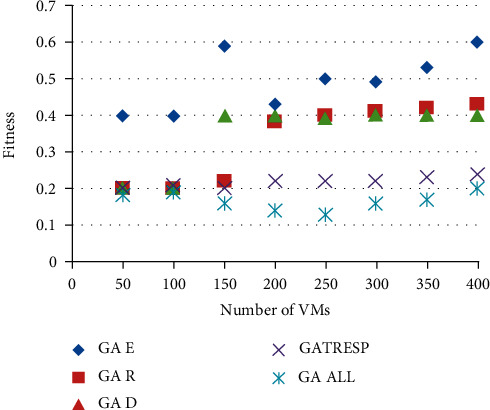
Comparison of fitness values between the 5 versions of GA.

Figures 6 and 7 show the results of the execution of the different GAs described above, using an increasing number of virtual machines to allocate. This therefore results from 8 executions of each of the 5 versions of the GA; the 8 executions correspond to the number of virtual machines to be placed, ranging from 50 to 400, on the 110 physical machines available. This variation in the number of virtual machines to allocate makes it possible to analyze the optimization results applied to a lightly loaded, moderately loaded, and heavily loaded system. The first figure (Figure 7) shows the results of the energy metric, and the second (Figure 8) shows the results of the time of *r* Response; then the results of the robustness metric are presented in Figure 9, and finally, Figure 10 presents the results of the dynamism metric.

Each of Figures 8to 10 contains five curves. They correspond to the five versions (GA *E*, GA *T*_Resp_, GA *R*, GA *D*, and GA All) of the genetic algorithm described above, with the number of virtual machines to be allocated on the *X* axis, and the value of the considered metric on the *Y* axis.

The analysis of these curves makes it possible to notice that on each of them, the curve representing the best result corresponds to the curve of the version of the GA which optimizes the metric concerned in Figure 10. This result is both logical and reassuring. In addition, it also allows to see the deviation of other GAs from the best solution. The most remarkable differences are given by the version of GA that only optimizes the energy metric. Indeed, if we look at the results of this GA for robustness or dynamism, it is undeniable that this version of GA (GA Energy) gives much worse results than the others. It also reinforces the idea that energy consumption is a very interesting parameter to study alongside these other two metrics. Moreover, in addition to the underlying environmental aspect in the optimization of this metric, it also allows to deduce that it is very relevant in this context of multiobjective optimization, being perfectly antagonistic with robustness and dynamism. Then, it is necessary to analyze the results of the GA All, optimizing in a fair way the four metrics. First of all, we can notice that this curve is never very far from the best curve in each of the figures. At first glance, this means that this version of the GA performs well overall for each of them. Unlike the other versions of GA studied, which automatically degrade one of the metrics, this version seems to be able to give fairly good results and find an interesting compromise so as not to disadvantage any QoS parameters. In order to be able to analyze and compare all the results of the GA All in relation to the others and therefore to better understand its performance and the compromises it generates, the last figure is proposed.

On this, we find on the abscissa the number of virtual machines to allocate, and the *Y* axis this time represents the fitness value of the objective function. This value is calculated by summing the normalized values of each metric. The applied normalization method uses the interval [min; max], corresponding to the smallest and the largest value obtained by the different versions of the GA for all the metrics and for the number of virtual machines given. Eight intervals are calculated, corresponding to the eight values of the *X* axis, and the normalized values are calculated as follows:

(6) $$\begin{array}{c} \vartheta_{cr}=\frac{M-\text{min}}{\text{max}-\text{min}}, \end{array}$$

with *M* the value of the metric. This normalization reduces all the values of the metrics between 0 and 1. Thus, for the metrics to be minimized, a value close to 0 represents a good result, and values close to 1 are worse. When a metric has to be maximized, such as dynamism, the interpretation of this value is reversed.

This normalization method was preferred in this study over the “Center-Reduce” method because the values of means and standard deviations used in the latter should have been calculated on a sample of five values, which is too little to have good precision.

Analysis of this comparison of the five versions of the GA shows that the multiobjective approach, represented by the GA All curve, achieves a better fitness value than all other versions of the GA. This indicates that by taking into account the four evaluated metrics, none of the GAs optimizing only one of them is able to obtain a better result than the GA All version, regardless of the system load represented by the gradual increase in the number of virtual machines to allocate.

## 7. Conclusion

This work explains the utilization of QoS modeling in a multilevel optimization approach. The quality of the selected service parameters, the modification of the virtual machines, and the DVFS are integral parts of the proposed planning problem. First, modeling was proposed by a genetic method. Dedicated to the problem of placing virtual machines in the cloud, this genetic algorithm has been implemented in two different versions. The former considers elementary services only, and the latter uses compound services. This version clearly complicates the resolution of the employment because a temporary feature must be integrated into the resolution. In fact, the terrain of a complex service, which is so simple, triggers the dependencies between services that the genetic algorithm must take into account to obtain the right investment solution. These two versions of the genetic algorithm are presented, and the two greedy algorithms, the round-robin and the best-fit sorted type, were used to allow comparison with the genetic algorithm. The characteristics of these two methods are given. It is important to note here that the version of the algorithm that uses the compound services is provided as preliminary work and requires future work.

## Figures and Tables

**Algorithm 1:**
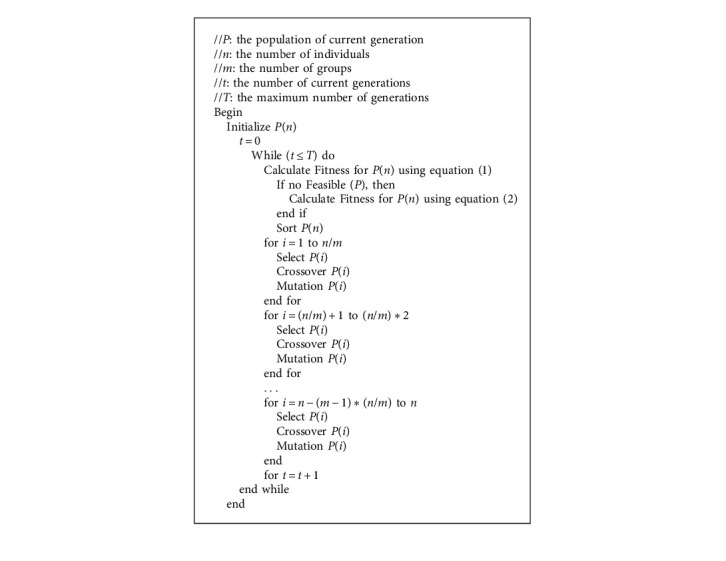
Basic genetic algorithm.

**Table 1:** Different parameters of the genetic algorithm.

Number of physical machines	110
Number of virtual machines	400
Number of individuals from the initial population	1500
Number of individuals in working population	120
Number of crosses	90
Number of mutations	120
Number of generations	600

**Table 2:** Universal setting for the use of an algorithm.

Working population individuals	30 to 50
Crossbreeding rate	Between 70 and 95%
Mutation rate	1 or 2%
Number of generations	Between 30 and 40

**Table 3:** Different versions of the GA associated with their optimization coefficients of each of the QoS metrics.

Name of GA	Coefficients applied to metrics			
	Energy	Time	Robustness	Dynamism
GA All	1	1	1	1
GA *E*	1	0	0	0
GA *T*_Resp_	0	1	0	0
GA *R*	0	0	1	0
GA *D*	0	0	0	1

## Data Availability

The datasets used and/or analyzed during the current study are available from the corresponding author upon reasonable request.
